# A Self‐Assembly Pipette Tip Restricted Access Mesoporous Polypyrrole Solid‐Phase Extraction Coupled With Capillary Electrophoresis With Diode Array Detection for the Determination of Enalapril in Urine Samples

**DOI:** 10.1002/elps.8126

**Published:** 2025-03-06

**Authors:** Iara Amorim Carvalho, Camilla Fonseca Silva, Keyller Bastos Borges

**Affiliations:** ^1^ Departamento de Ciências Naturais, Campus Dom Bosco Universidade Federal de São João del‐Rei São João del‐Rei Minas Gerais Brazil

**Keywords:** capillary electrophoresis, enalapril, polypyrrole, restricted access material, urine

## Abstract

A miniaturized self‐assembly pipette tip with restricted access mesoporous polypyrrole solid‐phase extraction, combined with capillary electrophoresis with diode array detection (CE‐DAD), was developed to rapidly extract and determine enalapril from urine samples. The CE‐DAD technique used 50 mmol L^−1^ phosphate (pH 7) as the background electrolyte, a voltage of 13 kV, a 30 mbar hydrodynamic injection for 4 s, a capillary temperature of 25°C, and a wavelength of 195 nm to achieve a migration time of 6.3 min with satisfactory peak asymmetry and no interfering and/or baseline noise. The factors that influenced the extraction efficiency were evaluated and optimized: 750 µL sample at pH 7.5, 40 mg adsorbent, 250 µL hexane as a washing solvent, and 750 µL acetonitrile as eluent, resulting in recoveries around 74%. Linearity was acceptable in the 100–3000 ng mL^−1^ range, and the selectivity and accuracy were also suitable. The limits of detection and quantitation were 30 and 50 ng mL^−1^, respectively. The adsorbent effectively removed 87% of the proteins and may be reused three times. The analytical approach was successfully verified and used to analyze enalapril in urine samples collected from volunteers. Finally, the greenness of the researched technique was assessed using five measures that showed good eco‐friendliness.

## 1 Introduction

Antihypertensives are a broad category of orally delivered medicines. They not only lower blood pressure but also help prevent other cardiac problems. There are several types of antihypertensive medications, which are represented by diuretics, adrenergic blockers, calcium channel blockers, direct vasodilators, and angiotensin‐converting enzyme inhibitors. Cardiovascular diseases are among the top causes of mortality globally. The failure to avoid and control risk factors exacerbates this illness. Patients with cardiovascular diseases often have other diseases such as hypertension, obstructive ischemic disease, hyperlipidemia, or diabetes. Therefore, these patients often need to take multiple medications [1, 2].

Captopril was the first angiotensin‐converting enzyme inhibitor registered, followed by enalapril, which was available for therapeutic use. Enalapril is a prodrug that, after oral administration, is absorbed and hydrolyzed into its active metabolite, enalaprilat. Enalapril has been used in the treatment of arterial hypertension, heart failure, reduction of proteinuria, and chronic kidney disease. Enalaprilat is an important component of the renin–angiotensin–aldosterone system. It leads to a decrease in the formation of angiotensin II and thus peripheral vasodilation, followed by a decreased secretion of aldosterone, causing less sodium and fluid retention. These two mechanisms lead to a decrease in blood pressure. Enalapril is rapidly absorbed orally, and peak plasma concentrations occur within 1 h. Peak concentrations of enalaprilat occur 3–4 h after an oral dose of enalapril. While enalaprilat has a half‐life of 11 h, enalapril has a half‐life of 1 h and is predominantly excreted by the kidneys. Urine is a commonly used biological matrix for analysis, because it is the main route of elimination for most drugs, its collection is easy and non‐invasive, it has high stability upon freezing, and it can be collected in large volumes [3].

Among the several methodologies, chromatographic and capillary electrophoretic methods have been investigated due to their ability to separate and measure many analytes in various matrices. Only a few methods were available for measuring enalapril and its metabolites in plasma, urine, and pharmaceutical formulations. These approaches employ gas chromatography (GC) with electron capture detection [4], high‐performance liquid chromatography (HPLC) with mass spectrometry (MS) or tandem MS (MS/MS) detection [5, 6, 7, 8, 9, 10, 11, 12, 13, 14, 15], UHPLC‐MS‐MS [16], ultraviolet (UV) detection [17, 18], and capillary electrophoresis (CE) with UV detection [19].

For the analysis of complex matrices such as urine, a sample preparation process is required to eliminate possible interferents that may interfere with the analysis and to pre‐concentrate the analyte [20]. Among the multitude of sample pre‐treatment procedures, such as protein precipitation and solvent extraction methods, adsorbent‐based approaches like solid‐phase extraction (SPE) have gained importance. SPE has various advantages, including better analyte recoveries, avoidance of emulsion formation, and reduced reagent and solvent consumption [21, 22]; besides, its potential for great selectivity, owing to the tunability of adsorbent characteristics, emphasizes its utility [23, 24].

The introduction of pipette tips SPE (PT‐SPE) has simplified the extraction process by allowing for simple solution aspiration and dispensing. This method has two major modes for aspirating and dispensing liquids. The first approach includes connecting an adsorbent‐loaded pipette tip to a syringe and using a plunger to aspirate and dispense the solution by pulling and pushing operations. This method provides tremendous pressure, allowing you to employ more tightly packed materials. Therefore, the development of porous adsorbents is important and a necessity, which makes selecting an appropriate adsorbent and its loading configuration within the pipette tip critical to optimizing the process [25, 26].

In recent years, researchers have focused on discovering new adsorbent materials with good properties, and polypyrrole (PPy)‐based materials have been gaining attention [27, 28, 29]. These materials offer good adsorption, low cost, and a large surface area. The adsorbent properties of PPy can be improved by adding a surfactant in the synthesis of polymer, which makes the material mesoporous, increasing the pore size by forming micelles [28, 29].

Therefore, in order to allow a better procedure, a mesoporous PPy (mPPy) double coated with hydrophilic monomers (HMs) and encapsulated with protein (bovine serum albumin [BSA]) has been synthesized and properly characterized. The adsorbent has two layers that can exclude macromolecules and extract enalapril from urine samples. PT‐SPE was optimized for different parameters. The PT‐SPE/CE‐diode array detector (DAD) system was validated and applied to real urine samples from volunteers taking enalapril. Finally, the greenness of the studied approach was evaluated using five metrics.

## 2 Experimental Section

### 2.1 Standard Solutions

Enalapril standard (100%) was obtained from Sigma‐Aldrich (St Louis, MO, USA). A stock standard solution of enalapril was prepared in HPLC‐grade methanol (J. T. Baker, Mexico City, Mexico) at 1 mg mL^−1^ and stored in an amber flask at −20°C, protected from light. Dilutions were prepared in methanol to obtain a solution at 40 µg mL^−1^ and in the range of 100 to 3000 ng mL^−1^ in aqueous (ultrapure water from Milli‐Q Plus, Belford, MA, USA) and urine samples.

### 2.2 Chemicals

Anhydrous monobasic sodium phosphate (Na_2_HPO_4_, 98%) was purchased from Neon (São Paulo, SP, Brazil), while sodium hydroxide was obtained from Synth (Diadema, SP, Brazil). Iron(III) chloride hexahydrate (FeCl_3_.6H_2_O) and ammonia hydroxide (NH_4_OH, 28%) were purchased from Dinamica (Diadema). Ethanol, methanol, and acetonitrile of HPLC grade were obtained from J. T. Baker. Glycerol dimethacrylate (GEMA), 2‐hydroxyethyl methacrylate (HEMA), sodium dodecyl sulfate (SDS), and pyrrole were purchased from Sigma‐Aldrich. BSA was purchased from Acros Organics (New Jersey, USA). Sodium borohydride and glutaraldehyde were obtained from Merck (Darmstadt, Hessen, Germany).

### 2.3 Instrumentation, Electrophoretic Conditions, and BGE Preparation

All CE analyses were performed using equipment from Agilent Technologies (Santa Clara, CA, USA), model CE 7100, consisting of an analyzer, an automatic sampler, and DAD operating at 195 nm. A fused silica capillary with a polyimide coating, having an internal diameter of 75 µm, a length of 59 cm, and an effective length of 50.5 cm, was used during the analyses. Before use, the capillary was conditioned by washing with 1 mol L^−1^ NaOH for 1 h and then with ultrapure water for 1 h. Every day before the analyses, conditioning was performed with 0.1 mol L^−1^ NaOH for 20 min. Between analyses, the capillary was washed with 0.1 mol L^−1^ NaOH solution for 2 min, with ultrapure water for 2 min, and finally with background electrolyte (BGE) solution for 3 min. After daily use, the capillary was washed with 0.1 mol L^−1^ NaOH and then with ultrapure water for 10 min. The optimized electrophoretic conditions were as follows: 50 mmol L^−1^ BGE phosphate, pH 9.0, adjusted with NaOH 0.1 mol L^−1^. The injection mode was hydrodynamic at a pressure of 30 mbar for 4 s, voltage of 13 kV, and temperature adjusted to 25°C. Every five analyses, the BGE was changed for method reproducibility. The BGE was prepared as follows: anhydrous sodium dihydrogen phosphate (0.2999 g) was weighed and dissolved in 50 mL of ultrapure water, and then the pH was adjusted to 9 with 0.1 and 1.0 mol L^−1^ sodium hydroxide.

### 2.4 Instrumentation for Materials Characterizations

Scanning electron microscopy (SEM) images were taken using a Hitachi Analytical Tabletop TM3000 microscope (Tarrytown, NY, USA) with an accelerating voltage of 20 kV and equipped with an energy‐dispersive spectrometer (EDS). All samples were applied to a carbon tape to obtain the SEM images. Quantax 70 software was used for elemental analysis. Transmission electron microscopy (TEM) images were obtained by electron microscope model JEM‐2100 (Jeol Ltd., Tokyo, Japan). ImageJ was used to measure particle size. Fourier transform infrared (FTIR) analyses were performed using a Shimadzu IRAffiny‐1 Fourier Transform Spectrometer (Kyoto, Japan) operating from 4000 to 400 cm^−1^ in the conventional KBr pellet method. X‐ray diffraction (XRD) analysis was performed on a D8 model Vinci Advance‐Bruker diffractometer with Cu‐Kα1 = 1.54059 Å and kα2 = 1.54443 Å radiation. The surface analysis was determined using Brunauer–Emmett–Teller (BET) and Barrett, Joyner, and Halenda (BJH) measurements utilizing the N_2_ adsorption–desorption technique at low temperature using Quantachrome's Autosorb iQ (Boynton Beach, FL, USA). Surface wettability has been shown by photographs obtained by a Nikon D90 Camera using 50 mm Nikon lens.

### 2.5 Adsorbents Syntheses

The synthesis is based on our previous work [27], in which RA‐mPPy‐HM‐BSA was obtained in three steps. First, mPPy was synthesized, followed by coating with HMs, GEMA, and HEMA, to obtain a restricted access material named RA‐mPPy‐HM. Finally, RA‐mPPy‐HM was encapsulated with BSA to obtain a double restricted access material denominated RA‐mPPy‐HM‐BSA. For this, initially, 13.5 g of FeCl_3_·6H_2_O was dissolved in 150 mL of ultrapure water. Subsequently, 0.4 g of SDS and 2 mL of pyrrole were added dropwise to the previous solution. The solution was kept under magnetic stirring for 3 h, and then the material (mPPy) was washed with methanol:ultrapure water (1:1, v/v) prior to being dried in an oven at 60°C for 4 h. Then, 1 g of mPPy was placed in a Falcon tube together with 0.930 mL of HEMA, 0.120 mL of GEMA, and 35 mL of chloroform. This suspension was sonicated for 10 min and dried at 60°C in an oven for 24 h to obtain RA‐mPPy‐HM. Finally, RA‐mPPy‐HM‐BSA, a double‐coated material, was obtained by coating with BSA. Briefly, 1.0 g of RA‐PPy‐HM was mixed in 20 mL of an aqueous solution of BSA (1%, m/v) prepared in 0.05 mol L^−1^ phosphate buffer (pH 6.0) and left to stand for 30 min. Then, the excess BSA was removed, and a crosslinking reagent was added (i.e., 5 mL of glutaraldehyde [25%]). This mixture was left to stand for 5 h. After this time, the excess glutaraldehyde was removed, and 10 mL of aqueous sodium borohydride solution (1%, m/v) was added. This mixture was left to stand for 15 min, and then the excess solution was removed. The final material (RA‐mPPy‐HM‐BSA) was dried in an oven at 50°C, washed with ultrapure water, and dried again.

### 2.6 Urine Samples

First, an aliquot of 10 mL of urine sample (blank samples) was added to a 15 mL flask. Then, 300 µL of 1 mol L^−1^ sodium hydroxide solution was added. This solution was then kept in a water bath for 1 h before being centrifuged at 2000 rpm for 3 min. The supernatant was collected and spiked with a standard solution of enalapril for sample preparation (Section 2.7) and procedure of method validation (Section 2.8). Similarly, urine samples at various pH levels were generated for examination in the optimization of sample preparation.

### 2.7 PT‐SPE Procedure

Urine samples were spiked with 40 µg mL^−1^ of enalapril, and some initial conditions was established to start the optimization of sample preparation, which were 20 mg of RA‐mPPy‐HM‐BSA, 500 µL of urine (without pH adjustment, ∼6.5), 500 µL of ultrapure water (washing solvent), and 500 µL of acetonitrile (elution solvent) (see Table S1). All analyses were performed in triplicate. Before adding the sample, RA‐mPPy‐HM‐BSA was conditioned with ultrapure water and methanol to activate the binding sites of the material. After each extraction, the eluent was collected, dried, and resuspended in 100 µL of 50 mmol L^−1^ BGE phosphate (pH 9.0):ultrapure water (9: 1, *v/v*) and analyzed by CE‐DAD.

In this way, different parameters such as of sample volume, sample pH, amount of adsorbent, washing solvent, volume of washing solvent, elution solvent, and volume of elution solvent were evaluated. After optimization, the reusability of the adsorbent was also evaluated, and the recovery of enalapril using RA‐mPPy‐HM‐BSA was compared with mPPy and RA‐mPPy‐HM. These analyses were also performed in triplicate.

$$\mathrm{Recovery}\;\mathrm{extraction}/\%=\frac{\mathrm{Experimental}\;\mathrm{value}}{\mathrm{Expected}\;\mathrm{value}}\;\times 100$$

The experimental value was determined from the electropherogram peak area or the analyte concentration found in spiked urine samples after the sample preparation procedure, and the expected value is calculated from the standard area or added concentration.

### 2.8 Method Validation

Validation process was performed based on limit of detection (LOD), limit of quantification (LOQ), selectivity, intra‐day and inter‐day precisions, accuracy, extraction recovery, stability (long‐term—2 years), robustness, and calibration linearity, all of which were evaluated to accomplish this validation.

### 2.9 Method Application

The Ethics Committee of the Federal University of São João del‐Rei approved the study protocol, which was followed in accordance with the Declaration of Helsinki (project identification code, CAAE number: 20839019.7.0000.5151). Each experiment was performed in accordance with any applicable rules or regulations. Before participating in the study, the participant also provided their informed consent for inclusion. In the application of the method, urine samples from volunteers were analyzed after oral administration of enalapril 20 mg (Renitec 20 mg).

## 3 Results and Discussion

### 3.1 Optimization of CE Condition

To achieve an acceptable CE condition, six factors were investigated, including the type, concentration, and pH of BGE, voltage, diameter, and size of the capillary, and temperature. Accordingly, between two basic BGEs, at the same concentration and pH 9, the phosphate buffer showed a stable baseline and good migration time in comparison to the triethylamine BGE. Then, other parameters were optimized, such as silica capillary with a 75 µm internal diameter, 59 cm length, and 50.5 cm effective length; a voltage of 13 kV; hydrodynamic injection of 30 mbar for 4 s; and temperature of 25°C. The detection wavelength was 195 nm. Under these conditions, it is possible to observe the enalapril peak with a good migration time (∼6 min) in the electropherogram shown in Figure S1.

### 3.2 Adsorbent Characterizations

**FIGURE 1:**
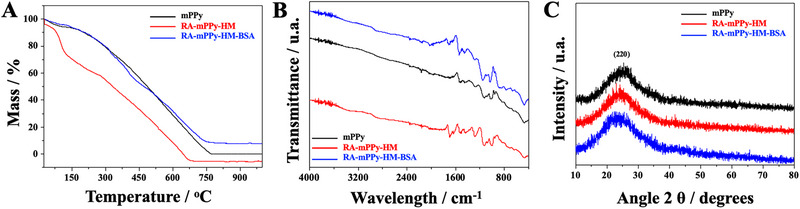
(A) Thermogravimetric analysis, (B) FTIR, and (C) XRD of mPPy (black line), RA‐mPPy‐HM (red line), and RA‐mPPy‐HM‐BSA (blue line).

The thermogravimetric analysis was performed to evaluate the thermal behavior and monitor the synthesis steps of the materials by measuring the mass variation as a function of temperature. Figure 1A shows the presence of three thermal events. The first event for the materials mPPy, RA‐mPPy‐HM, and RA‐mPPy‐HM‐BSA occurs due to a small mass loss (approximately 5%) up to 80°C. This can be attributed to the loss of moisture and/or decomposition of volatile reagents that were not consumed in the polymer synthesis. For mPPy and RA‐mPPy‐HM‐BSA, another thermal event can be observed occurring between 190°C and 660°C, resulting from the decomposition of the polymer. The material RA‐mPPy‐HM presents two more thermal events in addition to the initial mass loss. The second thermal event occurs between 80°C and 115°C, with a mass loss of approximately 20%, probably due to the presence of GEMA and HEMA on the surface of the material. The third thermal event occurs between 115°C and 660°C due to the decomposition of the polymer chain [30].

Figure 1B shows the FTIR spectrum, in which it is possible to analyze the characteristic functional groups of each material. The spectra of RA‐mPPy‐HM and RA‐mPPy‐HM‐BSA are similar to the spectrum of pure pyrrole. The bands at 1625 and 1550 cm^−1^ are associated with the conjugated C═C bond, which, together with the bands centered at 1450 and 1375 cm^−1^, are characteristic of the pyrrole molecule. The absorption band at 1087 cm^−1^ results from the plane deformation of the ═C─H bond and the N─H bond of the pyrrole ring. The peaks at 1288 and 1026 cm^−1^ correspond to the C─N stretching vibration and C─H deformation vibration of mPPy. At 1916 cm^−1^, the C─C deformation vibration out of the ring plane is found. Furthermore, the bands at 725 and 675 cm^−1^ confirm the presence of an unsaturated C═C bond [29, 31]. The ring deformations and out‐of‐plane C─H deformation are observed at 982 and 946 cm^−1^. It should be mentioned that the FTIR results are consistent with those found in the literature for PPy. Table S2 presents the main FTIR signals.

The crystallinity and purity of the phases of the synthesized materials were evaluated by XRD. In Figure 1C, it is possible to observe the diffraction peak at 2θ = 25.32, which is attributed to the crystal plane (220). The materials RA‐mPPy‐HM and RA‐mPPy‐HM‐BSA present the same pattern as pristine mPPy, with a broad peak at 2θ = 20°–30°, attributed to the repeating unit of the pyrrole ring. A broad band at the beginning of the diffractogram confirms the amorphous nature of the material, showing the low crystallinity of the polymer matrix [31].

**FIGURE 2:**
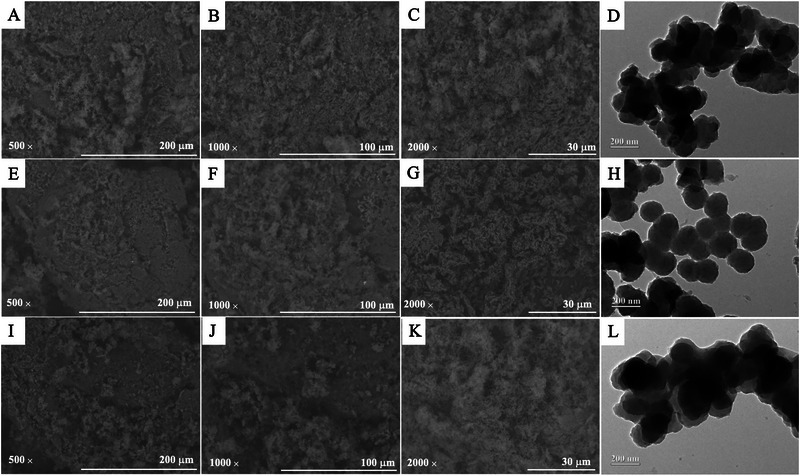
SEM images of mPPy: (A) 500×, (B) 1000×, and (C) 2000×; RA‐mPPy‐HM: (E) 500×, (F) 1000×, and (G) 2000×; RA‐mPPy‐HM‐BSA: (I) 500×, (J) 1000×, and (K) 2000×. TEM images of (D) mPPy, (H) RA‐mPPy‐HM, and (L) RA‐mPPy‐HM‐BSA.

SEM images were used to evaluate the surface morphological characteristics of the materials. The surface modifications of the materials were evaluated with HM coating on PPy and when BSA was added. Figure 2 shows the SEM (magnifications at 500, 1000, and 2000×) and TEM images of the three steps of synthesis. In SEM images, it is possible to observe an agglomerated material without defined shape and size, that is, without a homogeneous surface. It is evident that all materials have uneven surfaces, lack distinct sizes and shapes, and have a propensity to agglomerate particles. The adsorption capacity may be enhanced by the apparent spongy structure displayed by materials. The TEM images demonstrated that following coating with HM (Figure 2H) and encapsulating with BSA (Figure 2L), the apparent structural form in mPPy (Figure 2C) does not significantly alter. Figure 2L show the material encapsulated with BSA, that is, RA‐mPPy‐HM‐BSA tends to cluster more than RA‐mPPy‐HM, which presents itself as spherical particles [32]. The size of the particles and the structural form of the materials may be determined from TEM images. According to ImageJ, the diameter of the particles showed a range of sizes, from 120 to 190 nm, which was calculated by counting incompletely aggregated spheres at random.

The EDS technique allows a semi‐quantitative analysis of the chemical elements present in the materials, as can be seen in Table S3. For PPy, large amounts of carbon, nitrogen, and oxygen were observed, as well as small amounts of chlorine, sulfur, and aluminum due to some contamination. Cl was also present in this material, which may have occurred due to the presence of FeCl_3_.6H_2_O particles that did not react during the synthesis. The carbon may have appeared due to the carbon tape used during the analysis of the materials or it may be explained by the presence of this element in the PPy chain. There was also an increase in the amount of oxygen. Through the EDS analysis, it was possible to observe expected elements in each material, which confirms that the synthesis was efficiently carried out.

In order to evaluate the hydrophilic and hydrophobic properties of the adsorbents, it was necessary to observe the contact angle of a water droplet on the surface of the material. A surface is called superhydrophilic if the angle is less than 5°, hydrophilic when the contact angle is less than 90°, hydrophobic if the angle is between 90° and 150°, and superhydrophobic if the angle is greater than 150°. Figure S2A shows the image of the water droplet on the surface of mPPy, with a contact angle greater than 90°, indicating its low wettability and characterizing this material as hydrophobic. The coating of mPPy with HM can be observed in Figure S2B. In this case, the material becomes hydrophilic once the water droplet is fully dispersed on its surface. Already, Figure S2C shows RA‐mPPy‐HM‐BSA. BSA is a hydrophobic protein; hence, RA‐mPPy‐HM‐BSA increased its contact angle to become hydrophobic. From this information and other characterizations performed, it is possible to conclude that the adsorbent was efficiently double coated [29].

Measurements of N_2_ adsorption–desorption of RA‐mPPy‐HM‐BSA provided information such as surface area, pore size, and pore volume, as well as the adsorption–desorption isotherm profile shown in Figure S3. According to BET tests, RA‐mPPy‐HM‐BSA has a specific surface area of 7.54 m^2^ g^−1^. Furthermore, BJH investigations demonstrated that RA‐mPPy‐HM‐BSA is a mesoporous material with an average pore size of 42.1 nm and a volume of 0.07 cm^3^ g^−1^. Similar results have been reported in literature [21, 29]. According to the IUPAC classification [33], the adsorption isotherm curve is Type III, suggesting a mesoporous system (with pores ranging in diameter from 2 to 50 nm) with weak interactions, which means the adsorbent–adsorbate interaction is weaker than the adsorbate–adsorbate interaction.

### 3.3 PT‐SPE Optimization

**FIGURE 3:**
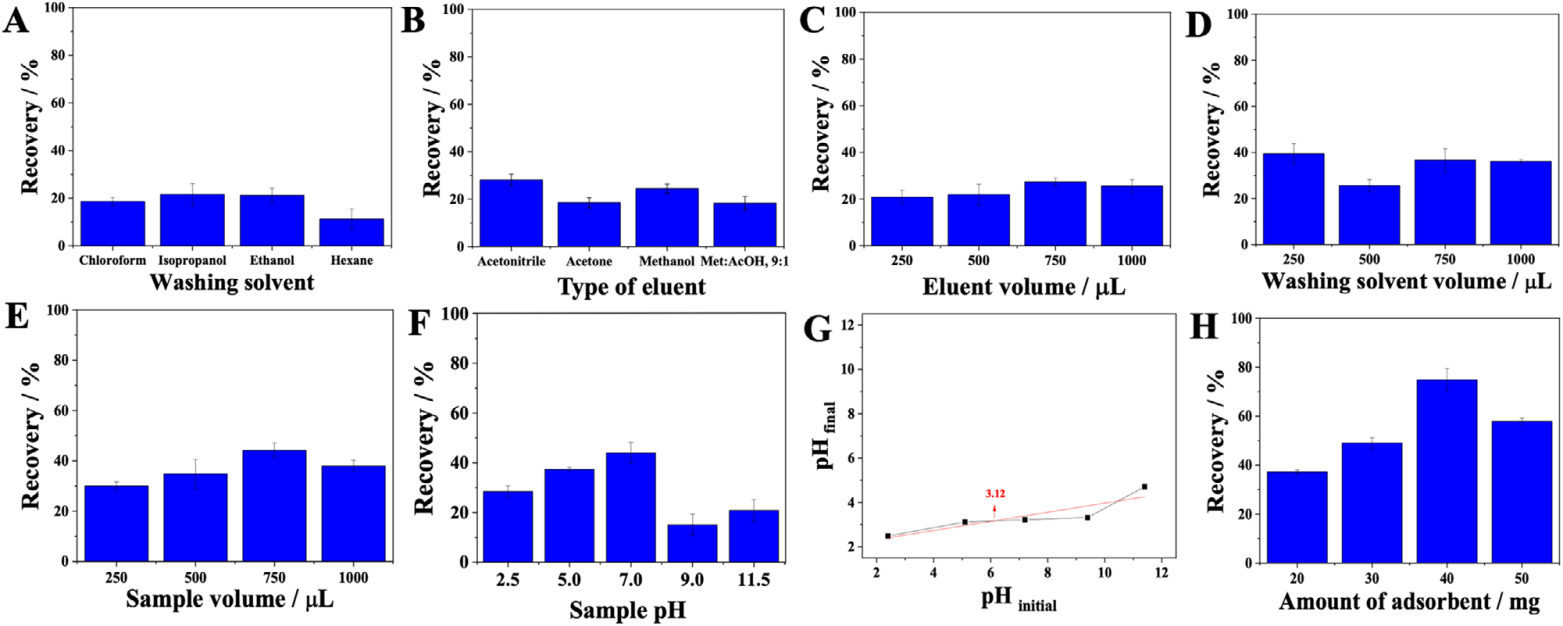
Evaluation of (A) washing solvent, (B) type of eluent, (C) eluent, (D) washing solvent volume, (E) sample volume, (F) sample pH, (G) point of zero charge, and (H) amount of adsorbent. All recovery extraction tests were performed on pooled urine samples.

Some initial conditions were established to start the PT‐SPE optimization of enalapril from urine samples (see Table S3). Initially, the washing solvent was studied, and the following solvents were evaluated: chloroform, isopropanol, ethyl alcohol, and hexane. The washing solvent should not solubilize the adsorbent. The ideal is one that eliminates the greatest amount of interferents and elutes the smallest amount of analyte. Analyzing Figure 3A, it can be seen that the best washing solvent was hexane.

The choice of elution solvent is very important in sample preparation process. The ideal elution solvent should be one that elutes the analytes more efficiently. The elution solvents evaluated were acetonitrile, acetone, methanol, and methanol:acetic acid (9:1, v/v). The best extraction recovery was achieved using acetonitrile (28.13%) (Figure 3B). This solvent probably has a polarity similar to the analyte polarity, which explains the improvement in recovery. Then, the elution volume was studied (250, 500, 750, and 1000 µL). The recovery improved slightly with the increase in the volume of the elution solvent, reaching a maximum of 28%. when 750 µL of acetonitrile was used (Figure 3C).

The washing solvent volume evaluated were as follows: 250, 500, 750, and 1000 µL. As washing solvent, the best washing solvent should elute the smallest amount of analyte. The volume of 500 µL of hexane was chosen (Figure 3D).

The sample volume studied were as follows: 250, 500, 750, and 1000 µL, and the results are illustrated in Figure 3E. It is possible to see that there is an increase in extraction recovery with the increase in sample volume, until reaching a volume of 750 µL of sample. Above this value, the saturation of the binding sites with possible interferents originating from the matrix occurs, impairing the extraction of the analyte. Thus, the volume of 750 µL was chosen, as it obtained a greater recovery of enalapril (44.19%).

The sample pH plays a very important role in the extraction of organic compounds from biological samples. In this way, it is possible to determine the state in which the compound is found, whether molecular or ionic, and therefore understand what type of interaction may be occurring between the adsorbent and the adsorbate. The range of 2.5 to 11.5 was used to evaluate the effect of pH (Figure 3F). Enalapril has three pKas: pKa_1_ = 3, pKa_2_ = 3.4, and pKa_3_ = 8. The calculated pH_PZC_ of RA‐mPPy‐HM‐BSA was approximately 3.25 (Figure 3G). If the pH of the solution is below the pH_PZC_, the surface of the adsorbent is positively charged, favoring the adsorption of anionic species. However, if the pH of the solution is above the pH_PZC_, the surface of the adsorbent will be negatively charged, favoring the adsorption of cationic species. At pH = 3, enalapril has a positive charge on the secondary amine, and more than 99% of the secondary amine is in the cation form. At pH ˂ 3, the carbonyl oxygen is partially protonated and the electrophilicity of the carbonyl group increases. On the other hand, at pH ≥ 7, the stabilizing effect of maleic acid is attenuated [34]. The higher adsorption efficiency at pH 7 can be explained by the fact that at this pH, the surface of RA‐mPPy‐HM‐BSA is negatively charged, pH > pH_PZC_. The high recovery at pH 7 can be attributed to the amine group (−NH−) in the enalapril structure that has become neutral [32]. At pH 7, the enalapril is neutral, and the RA‐mPPy‐HM‐BSA is negatively charged, so that they may be interacting through Van der Waals, hydrogen, or dipole–dipole interactions. As can be seen in Figure 3F, the recovery efficiency is dependent on pH and can be controlled by several types of interactions.

Adsorption occurs through the deposition of an analyte on the surface of an adsorbent. In this study, 20, 30, 40, and 50 mg of RA‐mPPy‐HM‐BSA were studied (Figure 3G). It can be observed that with the increase in the amount of adsorbent, there was an increase in extraction recovery. However, when using 50 mg of adsorbent, the recovery decreased. Therefore, 40 mg of RA‐mPPy‐HM‐BSA was chosen for this method.

### 3.4 Protein Exclusion Test

**FIGURE 4:**
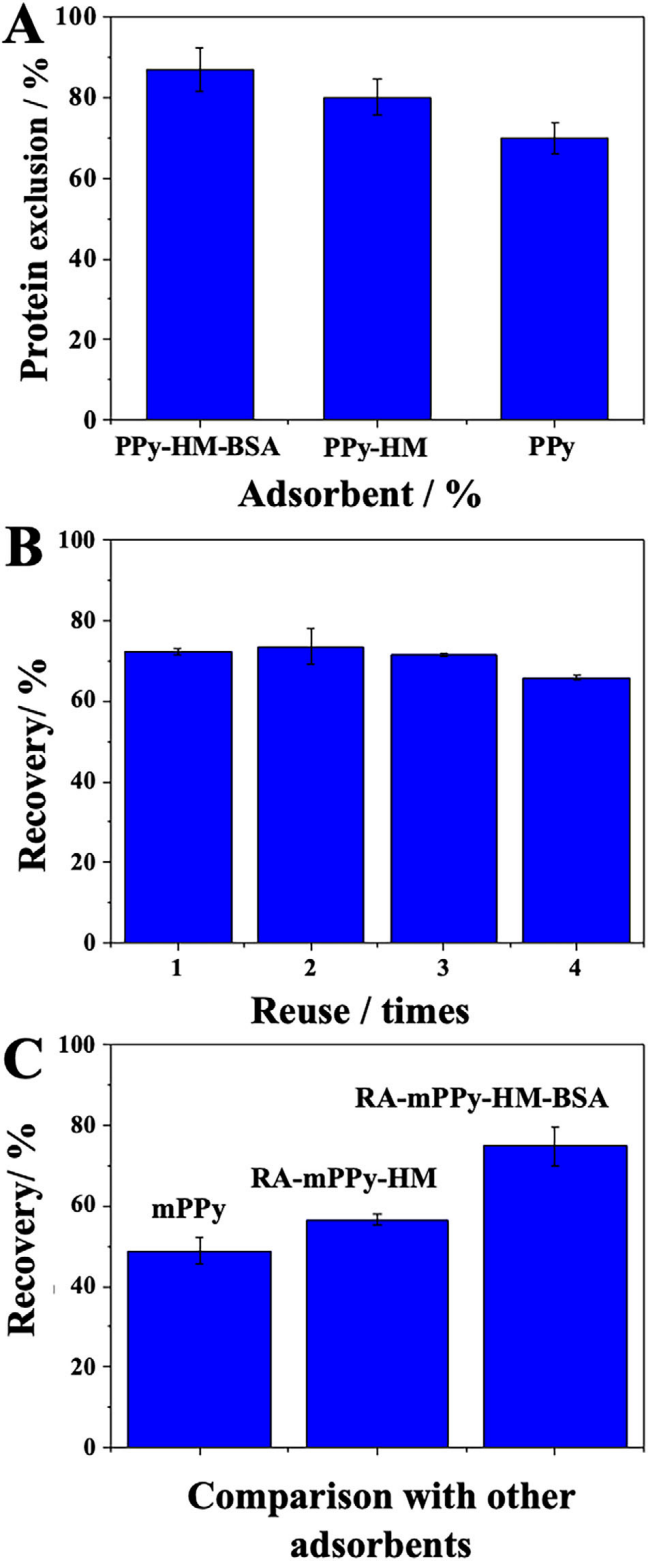
(A) Protein exclusion test, (B) reuse test, and (C) comparison of recovery of RA‐mPPy‐HM‐BSA with mPPy and RA‐mPPy‐HM.

The protein exclusion test was performed to evaluate the exclusion of macromolecules that are generally present in biological matrices, such as urine. To perform this test, 20 mg of each of the three materials (mPPy, RA‐mPPy‐HM, RA‐mPPy‐HM‐BSA) was weighed and placed in Falcon tubes containing 3 mL of BSA (0.1% m/v). Each tube was shaken for 1 min, with constant agitation of 2000 rpm using the vortex. After shaking, the solution was analyzed by UV‐VIS in a range of 200 to 400 nm. All tests were performed in triplicate and then compared with the BSA solution (0.1% m/v) that was not placed in contact with the adsorbent materials (signal for no adsorption = 100% exclusion). As can be seen in Figure 4A, mPPy, RA‐mPPy‐HM, and RA‐mPPy‐HM‐BSA excluded 70%, 80%, and 87% of the proteins, respectively. This result shows that as the coating was performed, the adsorbent gained a higher percentage of protein exclusion. RA‐mPPy‐HM‐BSA, with double coating, was the most efficient adsorbent for protein exclusion. Biological matrices such as urine have macromolecules, and coated materials would be better applied and would eliminate a greater amount of interferents present in these matrices.

### 3.5 Reuse

The reuse of RA‐mPPy‐HM‐BSA has been performed to evaluate the number of extractions that the same material can be subjected to without compromising the extraction efficiency. The optimized parameters for PT‐SPE are presented in Table S1. As can be seen in Figure 4B, the RA‐mPPy‐HM‐BSA material was subjected to four consecutive extractions. Between each analysis, the material was washed with 1 mL of acetonitrile and 1 mL of ultrapure water. The analyte recovery efficiency decreases after the third extraction. Therefore, it is recommended that the material be used only three times.

### 3.6 Comparison of RA‐mPPy‐HM‐BSA with mPPy and RA‐mPPy‐HM

The same optimized parameters obtained previously were used to evaluate the extraction recovery using mPPy and RA‐mPPY‐HM. The results are illustrated in Figure 4C. The adsorbent with the highest recovery capacity was RA‐mPPy‐MH‐BSA, demonstrating its efficacy and the coatings' efficiency in increasing extraction beyond protein exclusion. Certainly, the suggested adsorbent may be employed in PT‐SPE to extract enalapril from urine samples.

### 3.7 Method Validation

According to the parameters listed in the following sections, the PT‐SPE/CE‐DAD system was validated in accordance with the USFDA's bioanalytical system validation requirements [35].

#### 3.7.1 Linear Range and Sensitivity

To assess the linearity of PT‐SPE/CE‐DAD system, a set of seven concentration levels of urine samples were analyzed and used to generate the calibration plots. Linear regression analysis of the data set was conducted, linear fitting equations and their correlation coefficient were computed. The linearity of PT‐SPE/CE‐DAD for enalapril was evaluated using the correlation coefficients, as measures, within the specified concentration ranges, and it was also confirmed by the *F*‐test (ANOVA lack of fit), with calculated *F*‐value of 0.32 for enalapril below the value of *F* tabulated (2.64), in addition to a *p* value ≥ 0.05. A summary of the calibration parameters for enalapril is given in Table 1. The method was found linear in concentration ranges of 100–3000 ng mL^−1^.

**TABLE 1:** The analytical data for the determination of enalapril by PT‐SPE/CE‐DAD.

Linear equation^a^	Correlation coefficient (*r*)	Range (ng mL^−1^)	%RSD^b^
*y* = 0.0222*x* + 31.117	0.994	100–3000	8.80
**LOD (ng mL^−1^)**	**LOQ (ng mL^−1^)**	***F* value** ^c^	***p* value** ^d^
21.2	63.6	0.32	0.76

^a^ Calibration curves determined in triplicate (*n* = 3) for concentrations of 100, 500, 1000, 1500, 2000, 2500, and 3000 ng mL^−1^; *y* = *ax* + *b*, where *y* = peak area of the analyte, *a* = slope, *b* = intercept, and *x* = concentration of the measured solution (ng mL^−1^).

^b^ %RSD denotes relative standard deviation percentage of the slope of the calibration curve.

^c^ *F*_crit_ ≤ *F*_tab_ = 2.64.

^d^ *p* value ≥ 0.05.

The sensitivity was assessed and expressed as its limit of detection (LOD) and limit of quantitation (LOQ). The LOD and LOQ were calculated on the basis of signal‐to‐noise ratio approach, which give signal‐to noise ratios of 3.3 and 10, respectively. The LOD and LOQ values were found to be 21.2 and 63.6 ng mL^−1^, respectively. The LOQ value of enalapril demonstrates the adequate sensitivity of PT‐SPE/CE‐DAD system for the measurements of its concentration in urine samples of patients during therapy, as their reported concentrations [16].

#### 3.7.2 Selectivity

**FIGURE 5:**
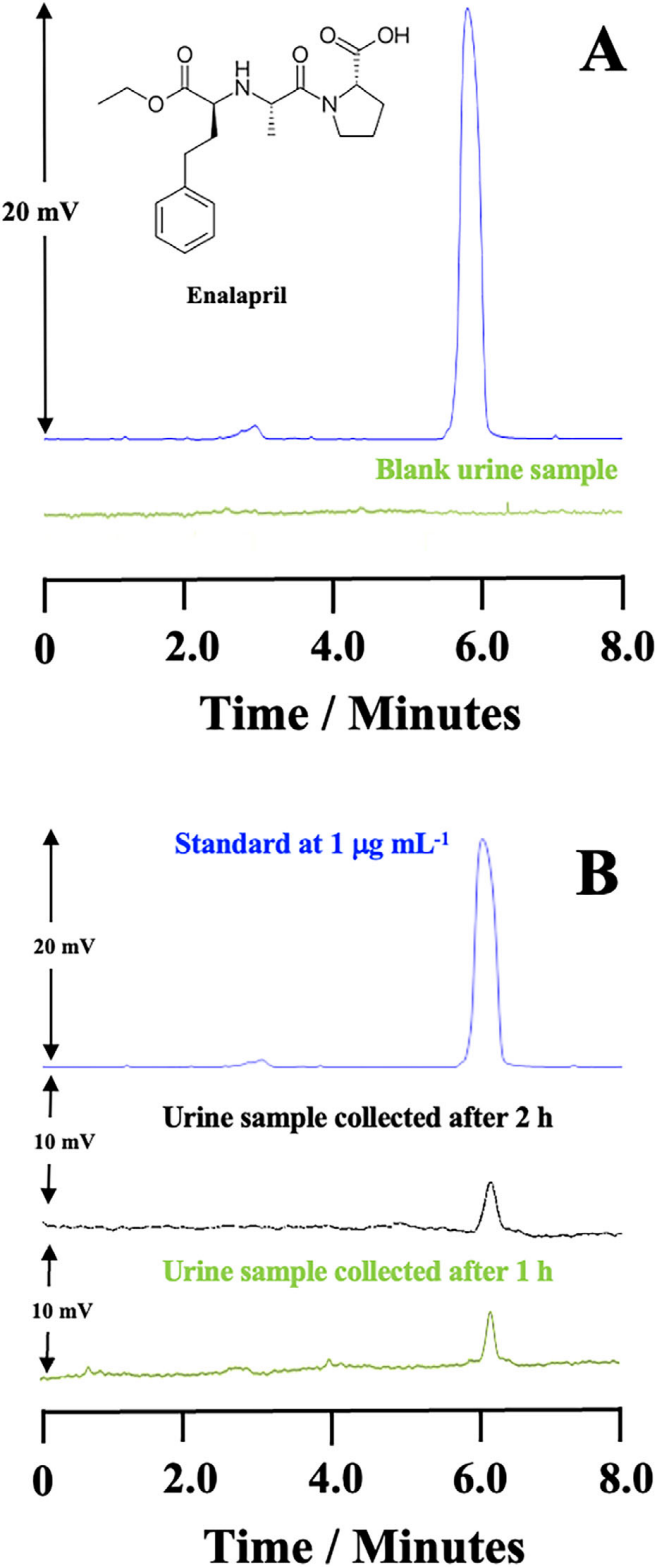
Electropherograms referring to (A) selectivity study and (B) method application for enalapril analysis employing PT‐SPE/CE‐DAD. Conditions: 50 mmol L^−1^ BGE phosphate, pH 9; hydrodynamic injection of 30 mbar for 4 s; voltage at 13 kV; detection wavelength at 195 nm; temperature at 25°C; and fused silica capillary with 75 mm internal diameter, 59 cm length, and 50.5 cm effective length.

The selectivity assessment was conducted by comparing electropherograms obtained from extracted urine samples (i.e., blank urine samples (pool) and spiked with enalapril at 40 µg mL^−1^). Human urine, being a complex matrix, can present different interferents that can disrupt the analytical process, so it is important to perform selectivity tests. The selectivity of an analytical method demonstrates the ability to detect and quantify an analyte even in the presence of interferents and impurities. The selectivity of a method presents its ability to differentiate and quantify the analyte in the presence of other components of the sample; urine, for example, contains interferents such as proteins [35]. Thus, it is possible to observe that during the migration times of the analyte, there is no presence of interferents that affect the analysis, which demonstrates the selectivity of the method for the enalapril analysis. The electropherograms (Figure 5A) demonstrate that there are no peaks from the urine matrix at the migration times of enalapril, confirming the selectivity of PT‐SPE/CE‐DAD system.

#### 3.7.3 Accuracy and Precision

Both intra‐day and inter‐day measures were evaluated for accuracy and precision. Enalapril at three concentration levels—low‐quality control (LQC, 500 ng mL^−1^), medium‐quality control (MQC, 1500 ng mL^−1^), and high‐quality control (HQC, 2500 ng mL^−1^)—was measured in six duplicates of spiked urine samples. In terms of percentage relative error (%RE), the intra‐day accuracy varied between −4.10% and 2.91%, while the inter‐day accuracy varied between −4.08% and 3.26% (Table S4). The percentage relative standard deviation (%RSD) was used to assess precision; intra‐day precision ranged from 0.44% to 1.62%, whereas inter‐day precision ranged from 1.09% to 2.21%. The PT‐SPE/CE‐DAD system's accuracy and precision were confirmed by the low values of %RE and %RSD, respectively.

#### 3.7.4 Stability

Several articles confirm that enalapril is stable under the conditions analyzed, as shown in Table S5. In this study, the urine samples were frozen at −20°C for 2 years, and after this period, it was possible to identify enalapril at the same concentration, thus proving its stability under freezing conditions.

### 3.8 Method Application

Urine samples from a volunteer were analyzed 1 and 2 h after the enalapril 20 mg was administered in order to apply the procedure. The purpose of this research was to demonstrate the proposed method's ability to detect and measure enalapril in urine samples. The electropherogram of an actual urine sample taken from a healthy volunteer following the administration of a 20 mg enalapril pill is displayed in Figure 5B. The concentrations of enalapril were 285.13 ng mL^−1^ in the second hour (black line) and 126.35 ng mL^−1^ in the first (green line). The results of this method are in line with previous studies that have been written up in the literature [16]. The PT‐SPE/CE‐DAD system may be used to identify levels of enalapril in complex matrices like urine, according to the investigations, which showed that the medication can be detected and quantified within 1 and 2 h. As a result, this approach is helpful in clinical practice for keeping an eye on high‐risk groups.

### 3.9 Comparison With Other Methods and Advantages

Table 2 shows the methods used to determine enalapril. It is also possible to find the analytical techniques applied, with LC‐MS‐MS being the most used. The matrix, sample preparation technique, recoveries, linear range, and LOQ/LOD are also described in the table. It is important to note that no study used a PPy‐based material employing PT‐SPE to extract enalapril from urine samples and determination by CE‐DAD.

**TABLE 2:** Literature review of analytical methods for the determination of enalapril.

Instrumental technique	Sample preparation	Matrix	Linear range (ng mL^−1^)	LOD/LOQ (ng mL^−1^)	Recovery (%)	Ref.
LC–MS‐MS	LLE	Plasma	0.10–100.0	LOQ = 0.1	67.8 ± 2.3	[6]
LC–MS‐MS	SPE	Plasma	0.064–431.806	LOQ = 0.064	85.4	[9]
LC–MS‐MS	SPE	Serum	1.61–206	LOQ = 1.61	75.92–88.69	[10]
LC–MS‐MS	SPE	Urine	11.6–12 000	LOQ = 11.5	96.5–103.8	[11]
LC–MS‐MS	SPE	Serum	0.2–200	LOQ = 0.2	77–104	[12]
LC–MS‐MS	Protein precipitation	Plasma	2–200	LOQ = 1.98	103.6	[13]
LC–MS‐MS	Protein precipitation	Plasma	1–200	LOQ = 1	87.73–112.96	[14]
HPLC–UV	Dilution	Tablet	16 000–48 000	LOD = 42.4	98.6–101.6	[15]
				LOQ = 129		
HPLC–UV	LLE	Plasma	6–30	LOD = 2.55	98.4–99.3	[18]
				LOQ = 6.25		
CE‐DAD	Dilution	Tablet	10 000–1 00 000	LOD = 2430	100.91	[19]
				LOQ = 7380		
CE‐DAD	PT‐SPE	Urine	100–3000	LOD = 21.2	74 ± 3.5	This work
				LOQ = 63.6		

Abbreviations: LLE, liquid–liquid extraction; PT‐SPE, pipette tip solid‐phase extraction; SPE, solid‐phase extraction.

The PT‐SPE/CE‐DAD has several distinguishing advantages: (i) the approach is cost‐effective and a highly efficient adsorbent; (ii) the innovative mesoporous adsorbent for PT‐SPE device is reliable, ensuring straightforward operation and consistent results, making it highly adaptable for use in diverse laboratory settings; (iii) the PT‐SPE is exceptionally rapid and efficient, capable of preparing samples in approximately 2 min; (iv) the subsequent CE‐DAD analysis can be completed in about 8 min, allowing the entire analytical process to be concluded in roughly 15 min; and (v) CE‐DAD can be considered a eco‐friendly instrumental technique.

### 3.10 Assessment of Method's Environmental Friendliness

Green analytical chemistry supports laboratory sustainability by reducing or eliminating the use of hazardous compounds, hence reducing their environmental and human health impacts. These methods are becoming more and more important in both industrial and scientific domains, because they can force and/or encourage laboratories around the world to develop greener procedures. Metrics that measure different aspects of environmental sustainability, such as AGREE (Analytical Greenness) [36], AGREEprep (Analytical Greenness metric for sample preparation) [37], SPMS (sample preparation metric of sustainability) [38], BAGI (blue applicability grade index) [39], and ComplexMoGAPI (Green Analytical Procedure Index) [40], are commonly used to evaluate the greenness of analytical methods.

**FIGURE 6:**
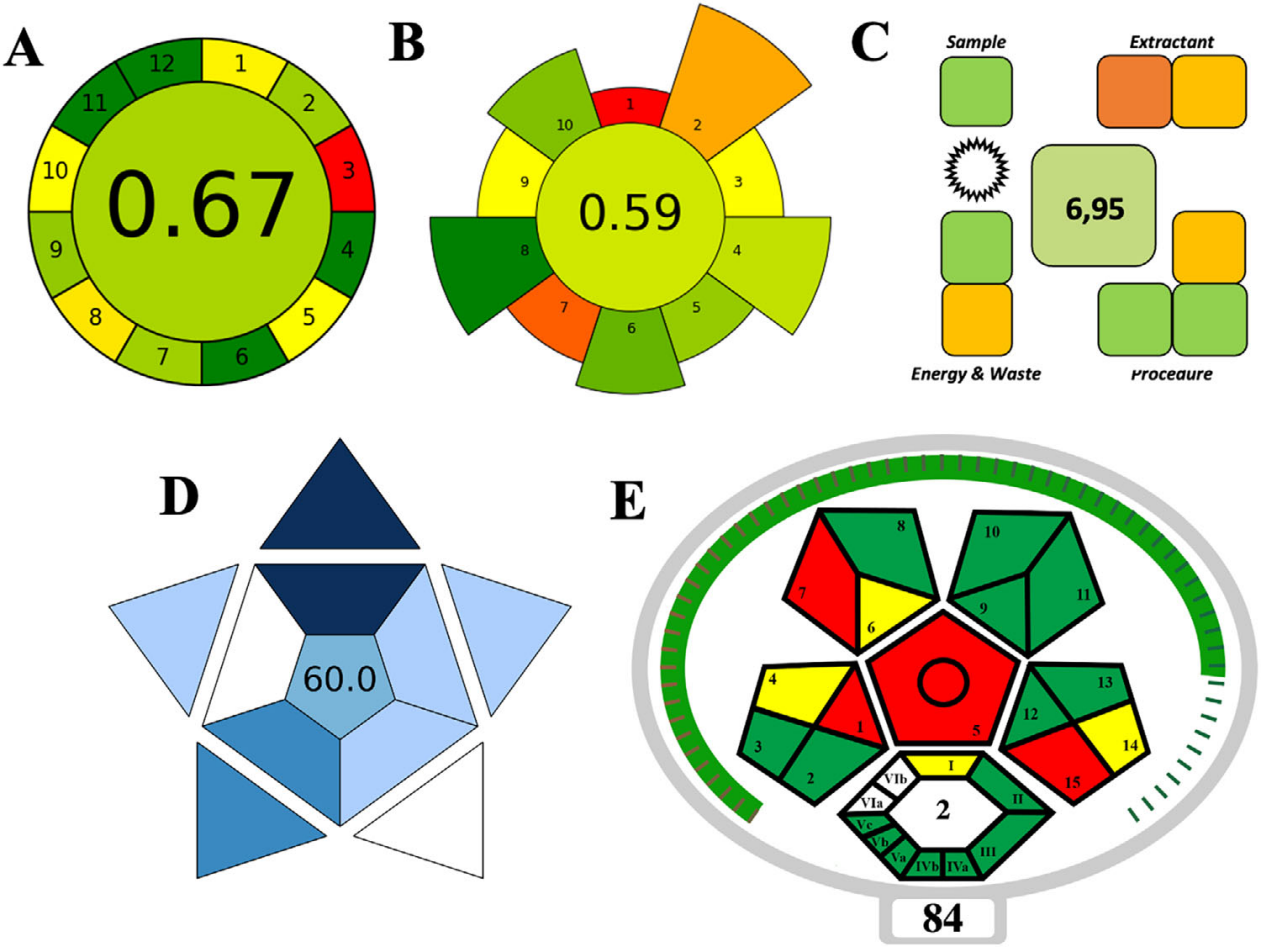
Metrics that measure environmental sustainability: (A) AGREE (Analytical Greenness); (B) AGREEprep (Analytical Greenness metric for sample preparation); (C) SPMS (sample preparation metric for sustainability); D) BAGI (Blue Applicability Grade Index); (D) ComplexMoGAPI (Green Analytical Procedure Index).

AGREE rates procedures according to how well they follow the 12 principles of green chemistry, in which higher scores indicate more environmental sustainability. With a score of 0.67 in the AGREE evaluation, the suggested approach demonstrated sufficient green performance, especially in terms of waste reduction, mainly toxic reagents and solvents (Figure 6A).

Furthermore, the primary purpose of AGREEprep was to evaluate how environmentally friendly sample preparation procedure was developed. The evaluation's outcome is displayed as a circular pictogram. The outside 10 portions and the inner circle's hues (green, yellow, and red) represent the overall and individual levels of greenness, respectively. As shown in Figure 6B, the total score of the proposed method was 0.59.

Likewise, the greenness of the sample preparation method was evaluated using SPMS. The hue of the clock‐like graphical report and the overall score equal to 6.95 indicated the green outcomes of the PT‐SPE procedure (Figure 6C).

Similarly, it is simple to utilize the BAGI to determine a method's strengths and weaknesses in terms of application and practicality. The degree of green was indicated by the pictogram's depth of blue. The number 60 in the pictogram's center was regarded as the analytical method's good greenness (Figure 6D).

ComplexMoGAPI evaluates the entire analytical process, including sample preparation, instrument operation, and waste management, offering a holistic view of a method's environmental impact. It employs a color‐coded system to visually represent the greenness level. In this assessment, the method demonstrated a commendable green profile, with good distribution of green, and a balanced yellow and red segments, showing a score of 84, indicative of its low environmental impact (Figure 6E).

## 4 Conclusions

In this work, we developed an analytical method for the determination of enalapril in urine samples. For this purpose, a restricted access polymeric material named RA‐mPPy‐HM‐BSA was synthesized to be used as an adsorbent in PT‐SPE. This material was properly characterized, proving that the synthesis was effective. RA‐mPPy‐HM‐BSA was able to exclude more proteins than the uncoated material, demonstrating that it can exclude a large part of the macromolecules present in a complex matrix. RA‐mPPy‐HM‐BSA showed good recoveries (∼74%). The linear range of the method was demonstrated to be between 100 and 3000 ng mL^−1^ (r > 0.99) with LOQ of 63.6 ng mL^−1^. Finally, the developed method was successfully applied to determine traces of enalapril in human urine. The method's environmental credentials were demonstrated by its eco‐friendliness, which uses small amount of material, solvents and samples, and low‐cost materials.

## Conflicts of Interest

The authors declare no conflicts of interest.

## Supporting information



Supporting Information

## Data Availability

The data that supports the findings of this study are available in the supplementary material of this article.
