# Supplementary material for: A Self‐Assembly Pipette Tip Restricted Access Mesoporous Polypyrrole Solid‐Phase Extraction Coupled With Capillary Electrophoresis With Diode Array Detection for the Determination of Enalapril in Urine Samples
Source: Electrophoresis. 2025 Mar 6;46(7-8):376–87. doi: 10.1002/elps.8126 (PMC12039168; doi:10.1002/elps.8126)
Supplement: Supplementary file 1 — Supporting Information [file ELPS-46--s001.docx]

**Supplementary Material**

**A self-assembly pipette tip restricted access mesoporous polypyrrole solid-phase extraction coupled with capillary electrophoresis with diode array detection for determination of enalapril in urine samples**

Iara Amorim Carvalho, Camilla Fonseca Silva and Keyller Bastos Borges*

Departamento de Ciências Naturais, Universidade Federal de São João del-Rei, Campus Dom Bosco, Praça Dom Helvécio 74, Fábricas, 36301-160, São João del-Rei, Minas Gerais, Brazil

*Corresponding author:

Prof. Keyller Bastos Borges, PhD, Departamento de Ciências Naturais, Universidade Federal de São João del-Rei, Campus Dom Bosco, Praça Dom Helvécio 74, Fábricas, 36301-160, São João del-Rei, Minas Gerais, Brazil

*e-mail: keyller@ufsj.edu.br

Phone number: +55 32 3379 – 5163

**Figures**


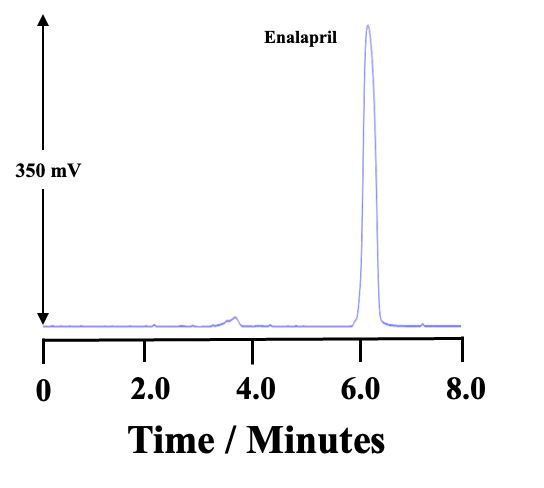


Figure S1. Capillary electropherogram referring to analysis of enalapril at 40 mg mL^-1^. Conditions: 50 mmol L^-1^ phosphate BGE pH 9, fused silica capillary with 75 mm of internal diameter, 59 cm of length and 50.5 cm of effective length, voltage at 13 kV, hydrodynamic injection of 30 mbar by 4 s and temperature at 25 ^o^C and wavelength at 195 nm.


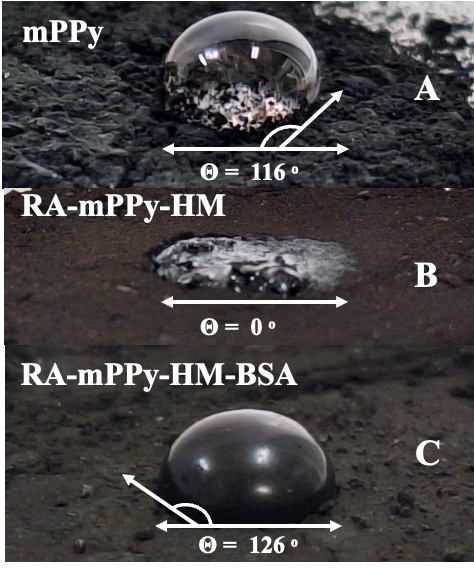


Figure S2. Wettability test for A) mPPy; B) RA-mPPy-HM; C) RA-mPPy-HM-BSA.


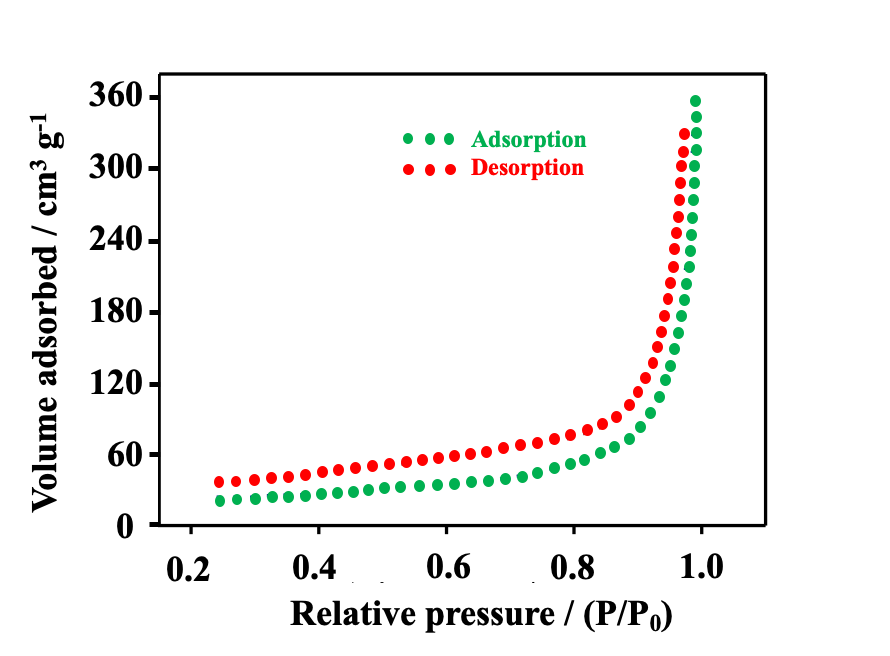


**Figure S3.** Nitrogen adsorption-desorption diagram for RA-mPPy-MH-BSA

**Tables**

**Table S1**. Optimized conditions for enalapril extraction using RA-mPPy-HM-BSA/PT-SPE.

| **Parameters evaluated** | **Initial conditions** | **Optimized condition** |
| --- | --- | --- |
| **Washing solvent** | hexane | hexane |
| **Washing solvent volume** | 500 µL | 250 µL |
| **Elution solvent** | acetonitrile | acetonitrile |
| **Elution volume** | 500 µL | 750 µL |
| **Sample volume** | 500 µL | 750 µL |
| **Amount of material** | 20 mg | 40 mg |
| **Sample pH** | 6.5 | 7.5 |
| **Enrichment factor** | 5 | 7.5 |
| **Recovery /%** | 23.6 | 74.5 |

**Table S2**. Main FTIR signals for mPPy, RA-mPPy-HM and RA-mPPy-HM-BSA.

| **Wavelength / cm ^-1^** | **Attribution** |
| --- | --- |
| 1625 / 1550 | Conjugated C=C bond |
| 1450 / 1375 | Asymmetric and symmetric C-C stretching vibrations |
| 1807 | Plane strain of bond =C-H |
| 1288 | C-N stretching vibration |
| 1026 | C-H deformation vibration of PPy |
| 1916 | Out-of-plane C-C deformation vibration of the ring |
| 725 / 675 | Unsaturated bond of the type C=C |
| 946 | Out-of-plane C-H deformation |

**Table S3**. Elemental composition of mPPy, RA-mPPy-HM e RA-mPPy-HM-BSA obtained by EDS.

| **Element** | **C** | **O** | **N** | **Cl** | **S** | **Al** | **Total** |
| --- | --- | --- | --- | --- | --- | --- | --- |
| **mPPy** | 69.45 | 11.50 | 14.60 | 2.76 | 1.49 | 0.20 | 100.00 |
| **RA-mPPy-HM** | 70.03 | 11.80 | 14.00 | 1.94 | 1.53 | 0.70 | 100.00 |
| **RA-mPPy-HM-BSA** | 68.38 | 15.04 | 13.53 | 1.30 | 1.16 | 0.59 | 100.00 |

**Table S4**. Precision and accuracy of the PT-SPE/CE-DAD for the determination of enalapril in urine

| **Nominal concentration / ng mL^-1^** | **500.0** | **1500** | **2500** |
| --- | --- | --- | --- |
| **Intra-day / n^a^= 6** | | | |
| **Analyzed concentration / ng mL^-1^** | 479.5 | 1544 | 2403 |
| **Precision / %RSD ^b^** | 1.62 | 1.44 | 0.44 |
| **Relative error / %RE ^c^** | -4.10 | 2.91 | -3.89 |
| **Inter-day / n^a^ = 3** | | | |
| **Analyzed concentration / ng mL^-1^** | 479.3 | 1549 | 2425 |
| **Precision / %RSD ^b^** | 1.86 | 1.09 | 2.21 |
| **Relative error / %RE ^c^** | -4.08 | 3.26 | -3.02 |

^a^ n = number of repetitions; ^b^ %RSD = mean relative standard deviation; ^c^ %RE = mean relative error.

**Table S5**. Stability of enalapril in biological matrices.

| **Matrix** | **Freeze-thaw cycles** | **Long term storage** | **References** |
| --- | --- | --- | --- |
| Plasma | 4 | 6 months at −30 °C | [9] |
| Urine | 3 | 1 month at −20 °C | [11] |
| Plasma | 3 | −80 °C for 30 days | [14] |
| Tablet | - | 25 °C by 72 h | [15] |
| Plasma | 3 | −20 °C by 24 h | [18] |
| Tablet | - | 4 °C by 7 days | [19] |
| Urine | 3 | 2 years at −20 °C | This work |
